# TNF-*α* increases human melanoma cell invasion and migration *in vitro*: the role of proteolytic enzymes

**DOI:** 10.1038/sj.bjc.6601257

**Published:** 2003-09-09

**Authors:** E Katerinaki, G S Evans, P C Lorigan, S MacNeil

**Affiliations:** 1 Section of Medicine, Division of Clinical Sciences, Northern General Hospital, Herries Road, Sheffield S5 7AU, UK; 2 University Division of Clinical Sciences (South), Unit of Child Health, The Children's Hospital, Sheffield S10 2SJ, UK; 3 Academic Department of Clinical Oncology, Weston Park Hospital, Sheffield S10 2SJ, UK

**Keywords:** melanoma, invasion, migration, TNF-*α*, MMP-2, degradative enzymes

## Abstract

Inflammatory mediators have been reported to promote malignant cell growth, invasion and metastatic potential. More specifically, we have recently reported that tumour necrosis factor alpha (TNF-*α*) increases melanoma cell attachment to extracellular matrix (ECM) substrates and invasion through fibronectin. In this study, we extend these investigations asking specifically whether the TNF-*α* effect on cell invasion and migration involves activation of proteolytic enzymes. We examined the effect of TNF-*α* on melanoma expression/activation of type IV gelatinases matrix metalloproteinases 2 and 9 (MMPs -2 and -9) and general proteolytic enzymes. Stimulation with TNF-*α* significantly increased both melanoma cell migration at 24 h (+21%) and invasion through fibronectin (+35%) but did not upregulate/activate the expression of latent MMP-2 constitutively produced by these cells and did not upregulate their general protease activity. However, the increased cell migration and invasion through fibronectin observed following stimulation with TNF-*α* were inhibited by the general protease inhibitor *α*_2_ macroglobulin. These findings suggest that the promigratory and proinvasive effect of TNF-*α* on this melanoma cell line may be mediated to some extent by induction of localised cell membrane-bound degradative enzyme activity, which is not readily detected in biochemical assays.

Proteolytic degradation of the basement membrane and the extracellular matrix (ECM) and movement through it are regarded as essential steps in the cascade of events leading to successful tumour cell invasion and metastasis. Studies on human melanoma have provided evidence that the plasminogen activator system and the matrix metalloproteinase (MMP) enzyme systems play an important role (Hofmann *et al*, 2000; Bodey *et al*, 2001); in particular, expression of MMP-2, an enzyme that degrades collagen IV in basement membranes, has been reported to increase in relation to malignancy of human melanocytic lesions (Väisänen *et al*, 1996).

Another important element of successful tumour progression is the generation of tumour stroma through induction of host's wound healing responses (Dvorak, 1986). Tumour stroma formation requires extensive tissue remodelling and is commonly characterised by an inflammatory cellular infiltrate consisting of macrophages, dendritic cells and lymphocytes (as reviewed by Balkwill and Mantovani, 2001).

This close resemblance of tissue remodelling during wound repair to tumour invasion and the fact that extensive host connective tissue ‘desmoplastic reactions’ are observed in invasive carcinomas but not around carcinomas *in situ* or benign tumours has led to the suggestion that the tumour infiltrating inflammatory cells and the cytokines they release may facilitate tumour cell growth, invasion and metastasis (Balkwill and Mantovani, 2001).

One of the inflammatory cytokines that has been associated with malignant tumour progression is TNF-*α*. A product of macrophages, TNF-*α* is one of the major mediators of inflammation that orchestrates a series of immune and stromal cell responses necessary for the destruction of damaged cells but also for the repair of tissues during wound healing (Beutler, 1999). Its production has been demonstrated in a number of human epithelial and haematological malignancies and it appears that both the protein itself and its receptor can be expressed in both the malignant and/or the stromal cells (Balkwill, 2002), suggesting potential autocrine and paracrine actions.

Studies on the effects of TNF-*α* using experimental models of invasion and metastasis have shown that it can often act as a tumour-promoting factor (reviewed by Balkwill, 2002). More specifically for melanoma, TNF-*α* has been reported to upregulate the expression of integrin subunits and the interaction of human melanoma cells with ECM substrates (Dekker *et al*, 1994; Zhu *et al*, 2002). In addition, the more recent study from this laboratory showed that TNF-*α* stimulated and alpha-melanocyte stimulating hormone (*α*-MSH) opposed cell attachment to ECM substrates and cell invasion through fibronectin, both of which would be consistent with TNF-*α* promoting metastasis and *α*-MSH providing partial protection against the action of TNF-*α*.

In the current study, we extend our investigations into the role of inflammation in melanoma invasion by studying the effects of TNF-*α* on melanoma cell migration and degradative enzyme activity.

Specifically, we examined the effect of TNF-*α* on melanoma cell general proteolytic enzyme expression and expression/activation of MMPs -2 and -9 and studied the extent to which degradative enzymes are involved in the TNF-*α* induced cell responses by looking at the effect of the general protease inhibitor *α*_2_ macroglobulin on TNF-*α* stimulated cell migration and invasion.

## MATERIALS AND METHODS

### Materials

Culture medium, additives and antibiotics were obtained from Gibco BRL (Life Technologies, Paisley, UK) and Sigma Chemicals Ltd. (Poole, UK). Foetal calf serum (FCS) was purchased from GlobePharm Limited (Esher, UK); newborn calf serum (NBCS) from Sigma Chemicals Ltd. (Poole, UK); trypsin/ethylenediaminetetraacetic acid (EDTA) and phosphate-buffered saline (PBS) tablets from Oxoid Ltd. (Basingstoke, UK). Human fibronectin was purchased from Sigma Chemicals Ltd. (Poole, UK); human recombinant TNF-*α* and *α*_2_ macroglobulin from Roche Diagnostics (Germany); and fluorescent BODIPY® casein (EnzChek® Protease Assay Kit-green fluorescence) from Molecular Probes Inc. (Eugene, USA). Tissue culture plastics were purchased from Corning Costar Corporation (Cambridge, USA). All other chemicals were of analytical grade.

### Culture of cutaneous melanoma cells

The human cutaneous melanoma cell line HBL was established in Professor Ghanem's laboratory from a lymph node metastasis of a nodular malignant melanoma (Ghanem *et al*, 1988). Cells were cultured in Ham's F10 (Gibco) medium supplemented with 5% FCS, 5% NBCS, 2 × 10^−3^ mol l^−1^ glutamine, 100 IU ml^−1^ penicillin and 100 *μ*g ml^−1^ streptomycin sulphate.

For the experiments where a 24-h preincubation was required, the cell culture medium was changed to RPMI-1640 medium (Gibco) supplemented with 20 ng ml^−1^ epidermal growth factor (EGF), 0.2% (w/v) D-glucose solution, 0.1% (w/v) bovine serum albumin (BSA), 2 × 10^−3^ mol l^−1^ glutamine, 100 IU ml^−1^ penicillin, 100 *μ*g ml^−1^ streptomycin sulphate and the relevant concentration of TNF-*α* was added. This serum-free medium was used for the fibronectin invasion assays as described later (serum-free invasion assay medium – SFIAM) and subsequently in all the assays where a 24-h preincubation period was required in order to keep the experimental culture conditions the same.

### Fibronectin invasion assay

A modification of the method we first described in Dewhurst *et al* (1997) was used to study the effect of TNF-*α* and *α*_2_ macroglobulin on the invasion of the melanoma cells through a layer of human fibronectin. Briefly, a suspension of 1.8 × 10^5^ HBL melanoma cells (contained in 500 *μ*l SFIAM with or without the appropriate drug concentration as required) was added to Nunc™ tissue culture inserts containing a polycarbonate filter with 8 *μ*m diameter pores covered with fibronectin (100 *μ*g ml^−1^). Cells were incubated for 20 h under standard culture conditions. At the end of the incubation period, the cells that had invaded through the filter (cells attached to the bottom of the well and to the undersurface of the filter) were collected separately from the cells that had not invaded (cells remaining attached to the upper surface of the filter). A combined analysis of the cell counts was used to calculate the percentage of the total population of cells that had invaded through the fibronectin monolayer.

When a preincubation period was required prior to the invasion assay, the cells were cultured in SFIAM with or without TNF-*α* 200 U ml^−1^ for the last 24 h before passaging and adding to the Nunc™ tissue culture inserts.

*α*_2_ macroglobulin was used as a general protease inhibitor after initially confirming its activity against trypsin prior to using it to inhibit proteolytic activity in the cell culture medium during the fibronectin invasion assay.

### Cell extraction of MMPs

Cell extraction of MMPs was performed when cell cultures were 70–80% confluent. Control and TNF-*α*-stimulated cells (incubated with TNF-*α* 200 U ml^−1^ for 24 h) were detached from the tissue culture flask and were lysed using cell extraction buffer (containing 50 mM Tris-HCl pH=7.4, 10 mM CaCl_2_, 0.05% Brij 35 and 0.25% Triton-X) for 15 min at 4°C. The cell conditioned medium was also collected for gelatin zymography analysis (following centrifugation at 2000 **g** for 5 min to remove any cellular debris).

### Cell extraction and measurement of general proteolytic activity using BODIPY® casein

For the purpose of extraction of general proteolytic activity, the melanoma cells were cultured in a 24-well plate at a density of 50 × 10^3^ cells per well with or without TNF-*α* (at serial dilutions ranging from 100 to 1000 U ml^−1^) for 24 h. At the end of the preincubation period, the conditioned medium was collected for measurement of proteolytic activity, and extraction from the cells was performed according to the protocol described above for extraction of MMPs.

General proteolytic activity was measured in cell conditioned medium and cell extracts using a quenched fluorescent BODIPY® casein assay (Molecular Probes Inc.). BODIPY® FL casein is a conjugate consisting of casein labelled with the fluorophore 4,4-difluoro-5,7-dimethyl-4-bora-3a, 4a-diaza-*s*-indacene-3-propionic acid (BODIPY® FL) (Jones *et al*, 1997). The casein is heavily labelled with the fluorescent BODIPY® FL dye, resulting in almost total quenching of the conjugate's fluorescence. The assay detects protease activity of a wide range of enzymes including serine proteases, sulphydryl proteases, acid proteases and metalloproteinases (Jones *et al*, 1997). The culture medium, or cell extracts, was added to an equal volume of BODIPY® casein at a concentration of 50 *μ*g ml^−1^ in a 10 mM Tris-HCl pH 7.8 assay buffer under low light conditions. Duplicate samples were added to round-bottomed 96-well plates and were incubated for 24 h at 37°C protected from light. The fluorescence emitted by the cleavage products of the quenched fluorescent substrate was proportional to the general proteolytic activity of the sample and was measured using a Denley Fluoscan platereader with an excitation filter at 480 nm and an emission filter at 530 nm. The results are expressed as arbitrary units of fluorescence following subtraction of background fluorescence in the appropriate control.

As an internal control for the assay, trypsin at a concentration of 3.3 *μ*g ml^−1^ was used in each experiment to demonstrate proteolytic degradation of BODIPY® casein. The above concentration was selected after initial experiments to confirm that the fluorescence emitted by the cleaved substrate was proportional to the concentration of trypsin. The following controls were also included in each assay: BODIPY® casein alone, fresh culture medium (SFIAM) with BODIPY® casein and extraction buffer with BODIPY® casein. Cell extracts were diluted 1 : 20 in assay buffer before being added to the fluorescent substrate as experimentation showed this to be the optimum dilution to minimise the effect of the extraction buffer itself on the fluorescent BODIPY® substrate.

### Sodium dodecyl sulphate–polyacrylamide gel electrophoresis gelatin zymography

Zymography was carried out on control (nontreated) and TNF-*α* treated cell extracts (24-h cell preincubation in SFIAM containing TNF-*α* 200 U ml^−1^ as described above) and conditioned medium (collected at the end of the 24-h preincubation period). Proteins were separated in nonreducing, nondenaturing conditions using a 10% resolving polyacrylamide gel containing 0.5% gelatin substrate. Internal human purified MMP-2 and MMP-9 standards (Biogenesis Ltd., Poole, UK) were included at concentrations of 20 ng per lane. After electrophoresis, the zymographs were developed, fixed and stained according to the protocol described by Baugh *et al* (1999).

### Migration assay

A modified ‘scratch wound’ migration assay was used for the assessment of the migration of the melanoma cells. The method was previously described by Cha *et al* (1996) for the study of the migration rate of keratinocytes.

Cells were plated in a 24-well culture plate in serum containing Ham's F10 culture medium at a density of 2 × 10^5^ cells per ml per well and were incubated for 24 h under standard culture conditions. After 24 h, when the cells had attached to the bottom of the tissue culture dish, the confluent cell monolayer was scratched with a plastic pipette tip to create a cell free zone in the well. The culture medium was removed and was replaced with an equal volume of fresh SFIAM with or without TNF-*α* 200 U ml^−1^ or *α*_2_ macroglobulin. The cells were incubated for a further 24 h under standard culture conditions. During this period, photographs of the scratched cell monolayer were taken at predetermined time points (0, 4, 8 and 24 h after the scratch was made) using an Apple McIntosh video microscope (Open Lab 3.0.4 software) and measurements of the distance between the two edges of the scratch were recorded for each different time point. The percentage reduction of the distance between the scratch edges at the different time points represented the migration rate of the melanoma cells. Triplicate wells were used for each experimental condition.

### Statistics

Control *vs* stimulated cell samples were analysed using Student's paired *t*-test for fibronectin invasion assays, migration assays and for general protease activity assays.

## RESULTS

### Effect of TNF-*α* on HBL melanoma cell invasion through fibronectin

Figure 1A

**Figure 1:**
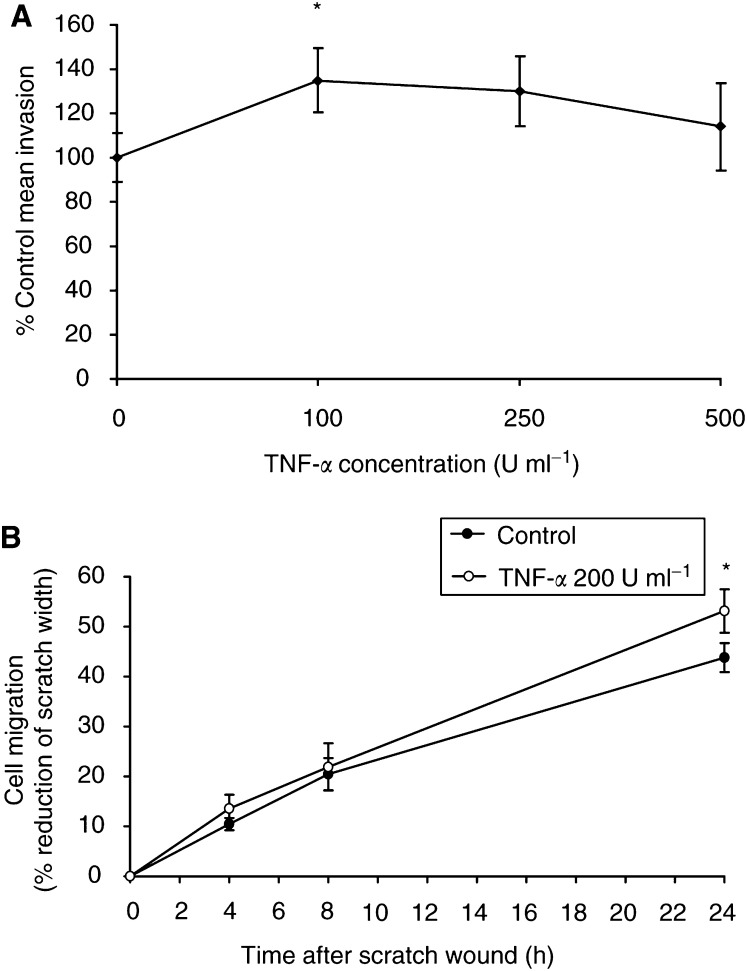
(**A**) Effect of TNF-*α* on HBL melanoma cell invasion through fibronectin. A significant increase in invasion (+35% above control) was observed with TNF-*α* at 100 U ml^−1^. Control invasion is expressed as 100%. Results are the mean±s.e.m. of four experiments and triplicate wells were used for each experimental condition in each experiment (^*^P<0.05). (**B**) Time course of TNF-*α* action on HBL cell migration on plastic as assessed by the ‘scratch wound’ migration assay. A significant increase in the overall migration at 24 h was observed for the cells stimulated with TNF-*α* at 200 U ml^−1^. The data shown are mean values±s.e.m. from *n*=8 experiments (^*^P<0.05).

illustrates the effect of TNF-*α* ranging from 100 to 500 U ml^−1^ on HBL melanoma cell invasion through fibronectin over 20 h. Unstimulated (control) cells showed a mean invasion rate of 24.6±2.7% (*n*=4 experiments). Stimulation with TNF-*α* 100 U ml^−1^ significantly increased the invasion rate of the cells to 33.3±3.5% (*P*<0.05), which represents an increase of +35% above the control level of invasion. Stimulation with higher concentrations of TNF-*α* (250 and 500 U ml^−1^) was less effective and the increases in invasion did not reach statistical significance.

To determine the optimum period of exposure for maximum response to TNF-*α*, cells were preincubated with TNF-*α* for 24 h prior to the invasion assay and were also subsequently stimulated with TNF-*α* again during the period of the invasion assay (a further 20 h). Preincubation with TNF-*α* resulted in a 43% increase in invasion above control level (*n*=3 experiments, *P*<0.05). Further addition of TNF-*α* to the invasion assay did not increase any further the invasiveness of these preincubated cells (results not shown). On the basis of these data, we concluded that the biological effects of TNF-*α* on invasion had occurred fully within 24 h.

### Effect of TNF-*α* on HBL cell migration

A ‘scratch wound’ model was used to examine the effect of TNF-*α* on melanoma cell migration. Cells stimulated with TNF-*α* 200 U ml^−1^ (cytokine present during the 24-h assay) had a significantly higher migration rate compared to the control (nonexposed to TNF-*α*) cells. The migration rate (represented by the percent reduction of the scratch width) of the TNF-*α* stimulated cells was 53.2±4.4% by 24 h, whereas the migration rate of the control cells at the same time point was 43.8±2.9%. This represented a 21% increase in migration as a result of TNF-*α* stimulation, *P*<0.05 based on eight experiments (as illustrated in Figure 1B). There were no changes in the morphology of the melanoma cells (control and TNF-*α* stimulated) at 8 or 24 h.

### Effect of TNF-*α* on melanoma MMP-2 and MMP-9 production and activation

**Figure 2:**
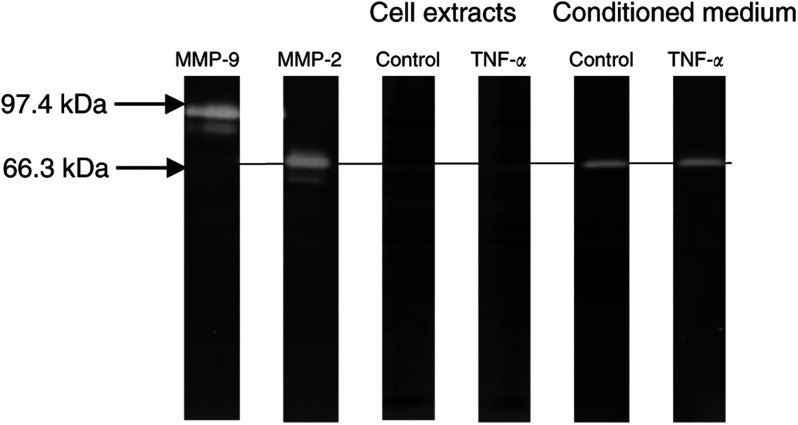
Representative gelatin zymograph of control and TNF-*α*-stimulated HBL melanoma cell extracts and cell conditioned medium. Cells constitutively expressed pro-MMP-2 (66.3 kDa band) in the culture medium and no upregulation or activation of the latent enzyme was observed after preincubation with TNF-*α* at 200 U ml^−1^. Lanes 1 and 2 represent internal human purified MMP-2 and MMP-9 standards.

HBL cells were incubated with TNF-*α* 200 U ml^−1^for 24 h prior to cell extraction and collection of cell conditioned medium for gelatin zymography (results are shown in Figure 2

). Unstimulated cells constitutively expressed MMP-2 in its latent form that was shed into the conditioned medium. No MMP-2 activity was recovered from the cell extracts.

No MMP-9 activity was detected in the cell conditioned medium or the cell extracts. Stimulation of cells with TNF-*α* did not result in any upregulation or activation of the pro-MMP-2 in the conditioned medium. Similarly, there was no evidence of TNF-*α* upregulating MMP-9 production in the cell extracts or the cell conditioned medium (again, as shown in Figure 2). Results shown are of a single experiment representative of data obtained in three different experiments.

### Measurement of general protease activity and validation of *α*_2_ macroglobulin activity

**Figure 3:**
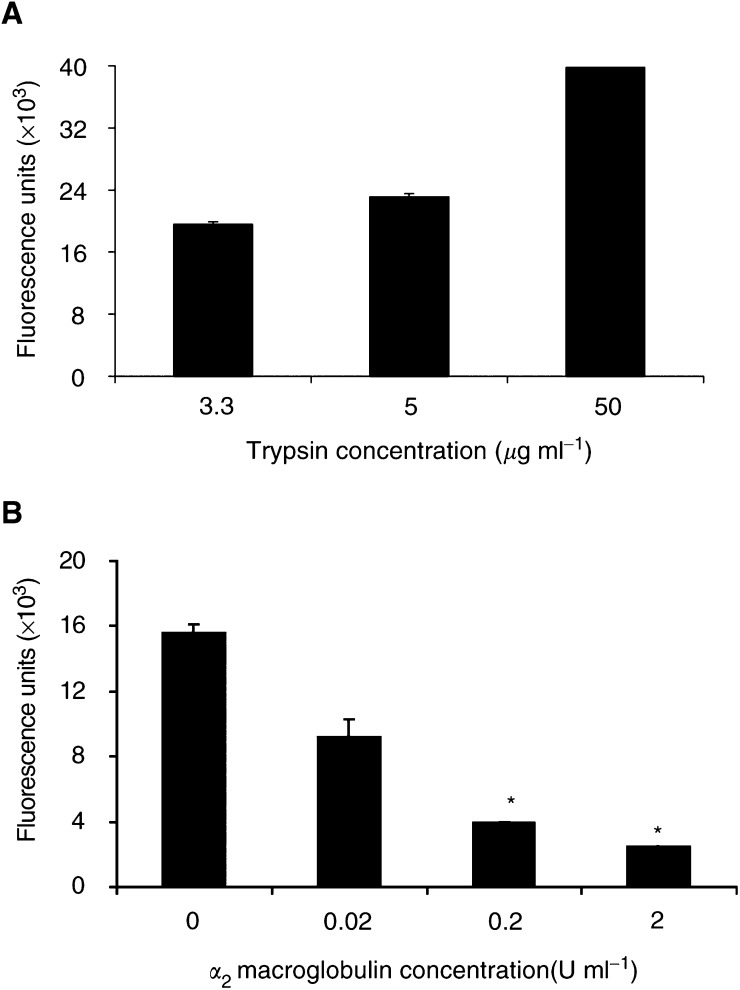
(**A**) The proteolytic activity of a series of trypsin concentrations was assessed by the quenched fluorescent substrate assay. Results shown are of one representative experiment. Duplicate wells were used for each trypsin concentration. (**B**) Inhibition of the proteolytic activity of trypsin (3.3 *μ*g ml^−1^) by the addition of the general protease inhibitor *α*_2_ macroglobulin. Inhibition of 84% was observed with *α*_2_ macroglobulin at a concentration of 2 U ml^−1^. Results show means±s.e.m. (*n*=2, ^*^P<0.05).

To confirm that the fluorescence emitted by cleavage of the BODIPY® casein substrate was representative of proteolytic enzyme activity, initial experiments used serial dilutions of trypsin (see Figure 3A

). From these, a concentration of trypsin was selected and increasing concentrations of *α*_2_ macroglobulin were added (see Figure 3B). The results show that significant inhibition was achieved with 0.2 and 2 U ml^−1^ *α*_2_ macroglobulin (*P*<0.05 based on *n*=2 experiments). *α*_2_ macroglobulin at a concentration of 2 U ml^−1^ was used for future experiments as this achieved 84% inhibition of trypsin activity.

### Effect of TNF-*α* on general protease activity of the HBL melanoma cells

Preincubation of cells with TNF-*α* at concentrations from 100 to 1000 U ml^−1^ for 24 h prior to cell extraction and conditioned medium collection, both of which were analysed, did not result in any upregulation of protease activity as shown in Table 1

**Table 1:** Effect of TNF-*α* on the general protease activity of HBL cells

	**Net fluorescence units**	
**TNF-*α* concentration (U ml^−1^)**	**Cell extracts**	**Cell conditioned medium**
0	5699±228	3776±118
100	6057±146	2726±759
250	4857±270	2908±771
500	6066±288	3929±532
1000	5114±744	2458±437

Cells cultured in a 24-well plate were stimulated with the relevant TNF-*α* concentration for 24 h prior to cell extraction and collection of cell conditioned medium for measurement of protease activity using the quenchedfluorescent substrate assay. Results are the mean±s.e.m. of two separate experiments.

. In general, protease activity recovered from the cell extracts was higher than that recovered from the conditioned medium possibly due to a large amount of intracellular proteolytic enzymes, including lysosomal stores of enzymes, being released during the cell extraction. Both, though, were of a similar log order and apparently unaffected by the addition of TNF-*α* to the cells for 24 h.

To measure noninvasively whether cell surface bound proteolytic activity was modified by TNF-*α*, we also added BODIPY® casein directly to the HBL melanoma cell culture medium during the period of cell invasion through the fibronectin monolayer in the invasion assay. Cell conditioned medium from the upper chamber (noninvading cells) and the lower chamber (invading cells) was sampled at the end of the 20-h assay period and the fluorescence of the sample, representing protease activity, was measured. TNF-*α* did not increase general protease activity in the conditioned media in these experiments (results not shown).

### Effect of *α*_2_ macroglobulin on HBL cell invasion

Although we had failed to show any effect of TNF-*α* on proteolytic activity of HBL melanoma cells, it was still conceivable that levels may have been beneath the level of detection of these biochemical assays and that proteases played some role in the invasion of these cells through fibronectin. Accordingly, we used *α*_2_ macroglobulin, an inhibitor of all classes of endoproteases, to examine its effect on control and TNF-*α* stimulated HBL melanoma cells. *α*_2_ macroglobulin inhibits proteases present in the cell conditioned medium or on the cell surface, but it cannot enter the cell or penetrate rapidly into the extracellular matrix due to its large size (720 kDa tetrameric glycoprotein).

**Figure 4:**
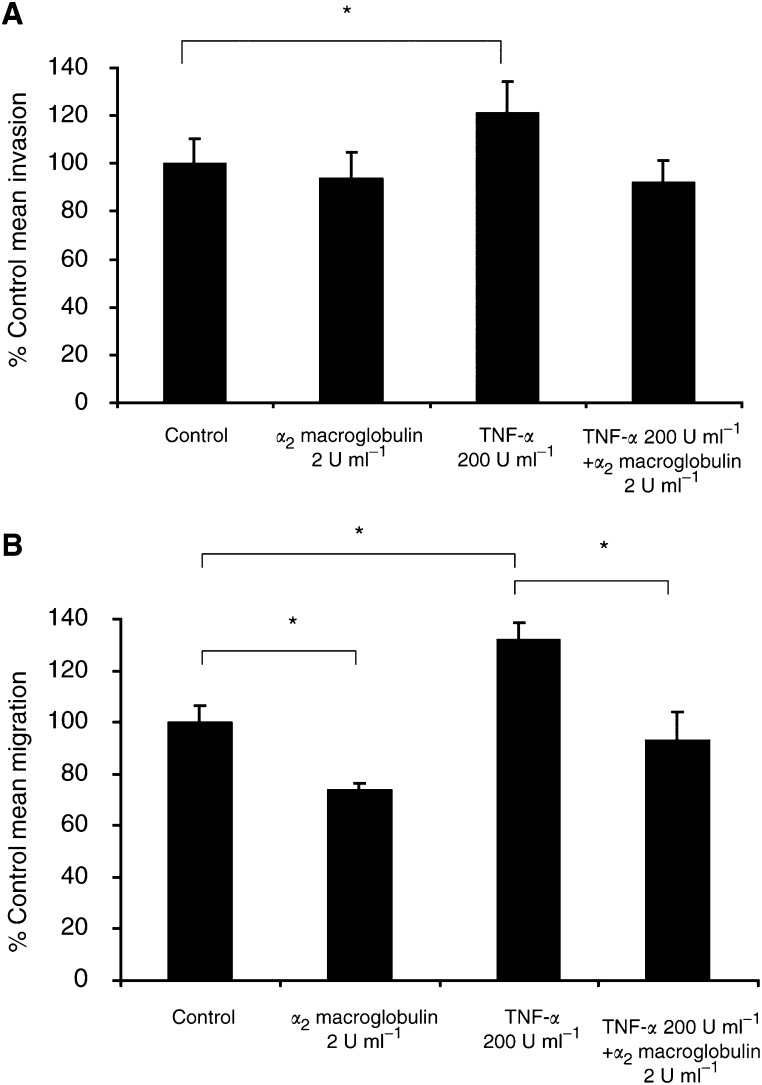
(**A**) Effect of the general protease inhibitor *α*_2_ macroglobulin at a concentration of 2 U ml^−1^ on HBL melanoma cell invasion through fibronectin. When added to non-TNF-*α*-stimulated cells, the invasion was not significantly reduced. In contrast, the increased invasion of the TNF-*α*-stimulated cells was completely inhibited by *α*_2_ macroglobulin and the invasion of these cells reduced to −8% of the control cell invasion. Results are the mean±s.e.m. of three experiments, ^*^P<0.05. (**B**) Effect of *α*_2_ macroglobulin (2 U ml^−1^) on the migration of control and TNF-*α*-stimulated cells. *α*_2_ macroglobulin decreased the control level of migration and the increase in migration resulting from cell exposure to TNF-*α* was completely inhibited by the protease inhibitor (*n*=3, ^*^P<0.05).

Using the model of fibronectin invasion, *α*_2_ macroglobulin (2 U ml^−1^) was added to the cell suspension in the upper chamber with or without TNF-*α* 200 U ml^−1^ for the 20-h culture period of the assay. Controls included cells with no stimulants and cells where only TNF-*α* was added during the invasion assay. In three separate experiments, *α*_2_ macroglobulin had no significant effect on cell invasion through fibronectin (−6% of the control level of invasion), as summarised in Figure 4A

. However, the significant increase (*P*<0.05) in invasion observed following stimulation with TNF-*α* (+21% of the control) was completely inhibited by the addition of *α*_2_ macroglobulin.

### Effect of *α*_2_ macroglobulin on HBL cell migration

*α*_2_ macroglobulin (2 U ml^−1^) was added to cells alone or in combination with TNF-*α* (200 U ml^−1^) for the 24-h duration of the migration assay. The migration rate of the non-TNF-*α* stimulated cells decreased by −26% (*n*=3, *P*<0.05) when *α*_2_ macroglobulin was added (see Figure 4B). The increase in cell migration seen on TNF-*α* stimulation (+32% above the control, *P*<0.05) was completely inhibited by the addition of the protease inhibitor (again as shown in Figure 4B).

## DISCUSSION

Inflammation represents the series of host responses triggered by infectious agents and traumatic (mechanical, thermal, chemical or radiation) insults. As such, it is invariably associated with the early phases of wound healing and persists to a certain degree, until regeneration of the injured tissues and restoration of function have been achieved. (Robins and Kumar, 1987).

Surgical excision of cutaneous malignant melanoma is undoubtedly a very strong stimulus of inflammatory responses within the skin as the wound healing process gets underway in the immediate postoperative period. Locoregional and distant melanoma recurrences can occur despite wide margin and complete tumour excision and well-conducted clinical studies have shown that there were no statistically significant differences in disease-free and overall survival rates when wide *vs* narrow margin excisions were compared (Lens *et al*, 2002). This phenomenon of locally recurrent tumours has led to speculations that some aspect of the excision itself has led to a ‘seeding’ of the wound bed with tumour cells that may have been present in the circulation but were apparently ineffective in establishing metastases until a wound was created.

To investigate this, animal studies have been undertaken which suggested that mediators released locally in the wound and systemically during the inflammatory phases of wound healing promote the growth, invasion and metastatic potential of human melanoma and other malignant tumour xenografts (Bogden *et al*, 1997; Hofer *et al*, 1998). There is also evidence from our laboratory that melanoma invasion in a model of human reconstructed skin requires the presence of host skin cells (namely fibroblasts and keratinocytes) that express characteristics of ‘wounded’ skin (Eves *et al*, 2000; Eves *et al*, in press). We recently reported that TNF-*α* upregulated integrin expression in a human melanoma cell line and also increased the ability of cells to bind to substrates and to invade through fibronectin (Zhu *et al*, 2002).

The current study continues this work asking the question of whether TNF-*α* promotes melanoma invasion through fibronectin via activation of melanoma proteolytic enzymes. In brief, there are two theories to explain how melanoma cells invade through ECM proteins. One requires that there is increased migration of melanoma cells along ECM proteins (Marshall *et al*, 1998), the other that melanoma cells degrade the matrix proteins to migrate through them (Bodey *et al*, 2001). In practice, it is almost certainly the case that both will occur *in vivo*.

In this study, we focused on the question of whether TNF-*α* was promoting melanoma invasion through fibronectin via upregulation of proteolytic enzymes. The first step in this investigation was to find the concentrations of TNF-*α* and the time course that was optimal for TNF-*α* upregulation of cell invasion through fibronectin and stimulation of migration. In practice, we found that TNF-*α* at a concentration of around 100–250 U ml^−1^ added for 20–24 h produced maximal effects on both cell migration and cell invasion. This result is consistent with previous reports from Professor Ghanem's laboratory that stimulation of HBL cells with TNF-*α* for 24 h resulted in a maximal increase in the expression of ICAM-1 (Morandini *et al*, 1998). Furthermore, previous work from our group on the effects of TNF-*α* on two human cutaneous melanoma cell lines, freshly isolated human ocular melanoma cells and melanocytes, has shown potent activation of transcription factor NF*κ*B within 30 min of TNF-*α* stimulation (at a concentration of 200 U ml^−1^) and significant inhibition by *α*-MSH in all the cell types examined (Haycock *et al*, 1999). More recently, we have demonstrated upregulation of integrin expression (specifically *α*_3_, *α*_4_ and *β*_1_ integrin subunits) within 24 h of TNF-*α* exposure for the HBL melanoma cells (Zhu *et al*, 2002) associated with TNF-*α* stimulated increased invasion through fibronectin – an effect largely blocked by *α*-MSH. Similar stimulatory effects of TNF-*α* were also seen on the invasion of three different ocular melanoma cell lines through fibronectin and MSH peptides blocked the response to TNF-*α* (Canton *et al*, in press).

Integrins are important cell surface signalling molecules expressed by normal and tumour cells and modulate processes such as attachment, motility and degradative enzyme (MMP) expression by the malignant cells (Jones and Walker, 1999). Upregulation of their expression has been associated with increased malignancy of melanoma cells (Marshall *et al*, 1998). TNF-*α* induced upregulation of integrin expression could be a critical step in the invasion of these cells.

Proteolytic enzymes have also been implicated in the process of melanoma cell invasion (Hofmann *et al*, 2000; Bodey *et al*, 2001). To investigate whether degradative enzyme upregulation is involved in the increased cell invasion observed with TNF-*α* stimulation, we examined the expression and activation of MMPs -2 and -9 using gelatin zymography, and the general protease activity of the melanoma cells using a quenched fluorescent substrate assay. As maximal upregulation of cell invasion was achieved with TNF-*α* between 100 and 250 U ml^−1^ and previous data from our group demonstrated maximal activation of NF*κ*B in these cells with 200 U ml^−1^ of TNF-*α* (Haycock *et al*, 1999), we decided to use TNF-*α* at a concentration of 200 U ml^−1^ for subsequent assays.

We were unable to demonstrate any increase to the expression of pro-MMP-2 constitutively expressed by these cells or any activation of the latent enzyme. Similarly, the general proteolytic activity expressed in the cell conditioned medium and by the melanoma cells themselves was not upregulated in response to preincubation with a wide range of concentrations of TNF-*α*.

However, although this result seems very straightforward, it is feasible that the levels of enzyme activity necessary for cell migration or cell invasion through fibronectin were already adequate for this purpose and that any increase in activity seen in response to TNF-*α* might just simply have been too small to be detected using these biochemical assays. It is possible that TNF-*α* may induce a localised pericellular, or cell membrane-associated, degradative enzyme activity sufficient for increased migration and invasion. This activity may not have been detected using the casein substrate.

For these reasons, we included the general protease inhibitor *α*_2_ macroglobulin. The large size of this protein precludes its access to intracellular proteases and its activity is restricted to the outer cell surface and extracellular space. With respect to invasion, *α*_2_ macroglobulin did not significantly reduce the basal level of invasion, but did inhibit the TNF-*α* stimulated increase in cell invasion. With respect to migration, *α*_2_ macroglobulin reduced both the basal and the TNF-*α* stimulated level of migration. This would be consistent with cellular migration involving some proteolytic activity, although it was evident that the extent of the inhibition was never more than about 26%. Thus, it is possible to hypothesise that membrane-bound or immediately pericellular proteolytic activity may be upregulated by TNF-*α*, allowing cells to migrate more effectively on ECM substrates or even matrix laid down by the cells themselves.

Other investigators have also recently reported upregulation of proteolytic enzymes by TNF-*α* in murine bone marrow cells and have also demonstrated increased *in vitro* degradation of bone collagen following TNF-*α* stimulation of osteoclasts that was successfully inhibited by the addition of a specific protease inhibitor (Katunuma *et al*, 2002).

It must be noted, however, that TNF-*α* can elicit a wide variety of biological responses in both normal and tumour cells (Balkwill, 2002). Some of these effects seem to be concentration dependent as high tissue concentrations can induce both cell necrosis and apoptosis (Balkwill, 2002). This effect is mediated via cell surface death receptors and it has been recently reported that stabilisation of these receptors in melanoma cells by cell exposure to the tissue inhibitor of metalloproteinases-3 (TIMP-3) sensitised these cells to apoptosis induced by TNF-*α* and other ligands (Ahonen *et al*, 2003).

The literature also reports that TNF-*α* may have complex effects on the regulation of degradative enzymes. Johnson and Baglioni (1990) have reported that stimulation with TNF-*α* induced expression of plasminogen activator inhibitor, type 2 (PAI-2) in SK-MEL-109 melanoma cells. This suggests that TNF-*α* may be a potent regulator of degradative enzyme activity during malignant cell invasion. More importantly, this study demonstrates that the TNF-*α* cell response can be modulated (both upregulated and downregulated) at a postreceptor level via activation of different second-messenger systems suggesting that the effect of TNF-*α* on various target cells can be modified inside the cell to produce the cell's final response to the cytokine.

Although our current study reports observations made on a single cell line, our data are in agreement with previous reports from the literature on the effects of TNF-*α* on the functional biology of different human malignancies including melanoma (Dekker *et al*, 1994; Balkwill, 2002) and extend ongoing work from our group on the effects of proinflammatory cytokines on human melanoma by examining the role of proteolytic enzymes in the invasion and migration of these cells.

In conclusion, we report that the proinflammatory cytokine TNF-*α* upregulates malignant melanoma invasion and migration *in vitro*. Taken together, the results of our current study combined with the data we have previously published (Zhu *et al*, 2002) suggest that TNF-*α* may exert its proinvasive effect on HBL melanoma cells via an integrin-dependent mechanism as well as a modest upregulation of degradative enzyme activity not readily detected in general protease assays.
